# Combining morphological and functional imaging parameters to diagnose primary bone neoplasms in the skull base, spine and sacrum

**DOI:** 10.1007/s00256-024-04742-z

**Published:** 2024-07-05

**Authors:** Vesna Miladinovic, Augustinus D. G. Krol, Johan L. Bloem, Judith V. M. G. Bovée, Suk Wai Lam, Wilco C. Peul, Ana Navas Cañete, Berit M. Verbist

**Affiliations:** 1 Department of Radiation Oncology, Leiden University Medical Center, Leiden, The Netherlands; 2 Department of Radiology, Leiden University Medical Center, Leiden, The Netherlands; 3 HollandPTC, Delft, The Netherlands; 4 Department of Pathology, Leiden University Medical Center, Leiden, The Netherlands; 5 University Neurosurgical Center Holland, Leiden University Medical Center, Leiden, Zuid-Holland Netherlands

**Keywords:** Bone neoplasms, Chordoma, Chondrosarcoma, MRI, CT, Functional imaging

## Abstract

**Purpose:**

Morphological magnetic resonance (MR) and computed tomography (CT) features are used in combination with histology for diagnosis and treatment selection of primary bone neoplasms. Isolated functional MRI parameters have shown potential in diagnosis. Our goal is to facilitate diagnosis of primary bone neoplasms of the skull base, mobile spine and sacrum, by a comprehensive approach, combining morphological and functional imaging parameters.

**Materials and methods:**

Pre-treatment MR of 80 patients with histologically proven diagnosis of a primary bone neoplasm of the skull base, mobile spine and sacrum were retrospectively analyzed for morphological and functional MRI parameters. Functional parameters were measured in 4 circular regions of interest per tumor placed on non-adjacent scan slices. Differences in values of functional parameters between different histologies were analyzed with Dunn’s test.

**Results:**

Chordomas were the predominant histology (60.0%). Most neoplasms (80.0%) originated in the midline and had *geographical* (78.2%) bone destruction. *Amorphous*-type calcification (pre-existing bone) was seen only in chordomas. Homogeneous contrast enhancement pattern was seen only in chondrosarcoma and plasmacytoma. *Ktrans* and *Kep* were significantly lower in both chordoma, and chondrosarcoma compared to giant cell tumor of the bone (p = 0.006 – 0.011), and plasmacytoma (p = 0.004 – 0.014). Highest diffusion-weighted MRI apparent diffusion coefficient (ADC) values corresponded to chondrosarcoma and were significantly higher to those of chordoma (p = 0.008).

**Conclusion:**

We identified the most discriminating morphological parameters and added functional MR parameters based on histopathological features that are useful in making a confident diagnosis of primary bone neoplasms in the skull base, mobile spine and sacrum.

## Introduction

Radiological diagnosis of benign, intermediate and/or malignant primary bone tumors of the axial skeleton can be challenging [1, 2]. By combining imaging with histopathology, a final diagnosis is reached, based on which treatment consisting of surgery, radiotherapy, and/or chemotherapy is planned. Various computerized tomography (CT) and functional, as well as morphological, magnetic resonance imaging (MRI) features assisting in diagnosis have been reported [3–9]. These reports contribute to improving diagnosis; however, their weakness lies in small series of these (rare) tumors (0.008—6.63 cases per 1 million person-years), and in using only a few selected parameters out of the plethora of imaging features [1]. Secondary to technical advances, the number of imaging parameters have increased over the years. Especially, functional MRI, such as dynamic contrast-enhanced (perfusion and permeability), and diffusion MR imaging have shown their potential in contributing to tissue characterization, which served as inspiration for its application in diagnosis [10–17]. Analyzing their relationship with histological characteristics, such as cellularity, angiogenesis and presence of mucoid, and combining them with traditional characteristics, including origin and extension, opened a window of opportunity to suggest a more accurate diagnosis in a non-invasive and low-risk manner.

Our goal is to diagnose primary bone tumors of the skull base, mobile spine and sacrum in a comprehensive approach, by combining morphological and functional imaging parameters.

## Materials and methods

### Patients

Electronic medical records of patients histopathologically diagnosed with primary bone tumor of skull base, mobile spine and sacrococcygeal region between January 2012 and December 2021, available at our tertiary bone tumor center database of the Leiden University Medical Center (LUMC), were reviewed. This retrospective research was approved by the local medical ethics committee and signed informed consent forms were obtained from all included patients.

**Fig. 1:**
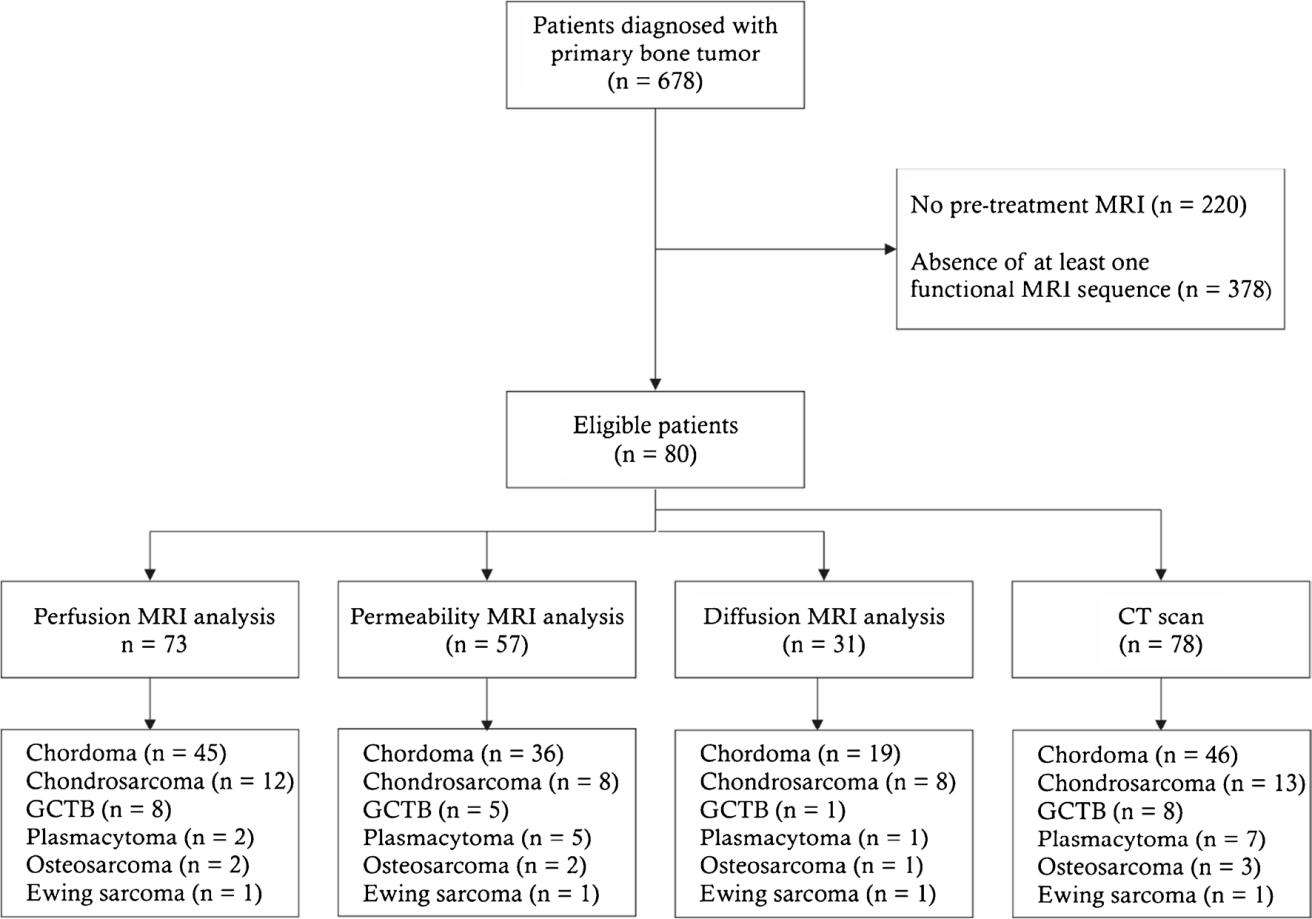
STRAD *( Standards for Reporting of Diagnostic Accuracy*) diagram of patient flow

Inclusion criteria were availability of pre-treatment MRI with at least one functional sequence, i.e., dynamic contrast enhanced (DCE) and/or diffusion weighted MRI. When available, pre-treatment CT scan was also analyzed. Of the 678 patients with primary bone tumors, 220 were excluded due to the absence of pre-treatment MRI and, 378 due to the absence of any functional MRI sequence. A total of 80 patients were included in this retrospective study. All of them had an MRI, which included a dynamic contrast-enhanced MRI in 73, allowing perfusion analysis, while 57 of these also allowed permeability analysis. Diffusion scans were available in 27 patients. Pre-treatment CT scan was available for analysis in 78 patients. Diagram of patient flow is presented in Fig. 1.

### Imaging

#### Protocols

CT scans (Toshiba, Aquilion ONE, Tokyo, Japan) were acquired with 120kVp energy setting, 3 mm slice thickness and 0.5 s rotation time.

MRI was performed on 1.5 T and 3.0 T scanners (Ingenia, Philips, Best, Netherlands) using a basic protocol consisting of: survey fast field echo (FFE) in three orthogonal plains; sagittal and transversal T1-weighted turbo spin echo (TSE), with repetition times (TR 600-750 ms), echo times (TE 6-20 ms); transversal T1 map with flip angles (FA) 5 and 15 (TR 3.2–3.4 ms), (TE 1.62–1.72 ms); sagittal and transversal T2 TSE mDixon with (TR 3000-4100 ms), (TE 6-60 ms); post-contrast: transversal dynamic T1 turbo field echo (TFE) with (TR 3.2–3.4 ms), (TE 1.62–1.72 ms). For skull base lesions we added to this basic protocol: transversal fluid-attenuated inversion recovery (FLAIR) TSE with (TR 11000 ms), (TE 125 ms), inversion time (TI 2800 ms); transversal DWI TSE (b = 0–800 s/mm^2^) with (TR 9600 ms), (TE 52 ms); post-contrast: sagittal and transversal T1 TSE mDixon with water, fat, and in phase reconstructions, (TR 700-820 ms), (TE 10-20 ms). For spinal and sacral lesions, we added to the basic protocol: transversal DWI IR-SE-EPI (b = 50-1000 s/mm^2^) with (TR 7200 ms), (TE 80 ms), (TI 220 ms); post-contrast: sagittal, transversal and coronal T1 TSE SPIR with (TR 650-700 ms), (TE 15-20 ms). Gd chelate contrast agent (Dotarem®, Guerbet, Villepinte, France) was administrated using a Medrad power injector with 2 mL/s flow at 0.2 mL per kilogram of bodyweight.

### Morphological and functional assessment

**Fig. 2:**
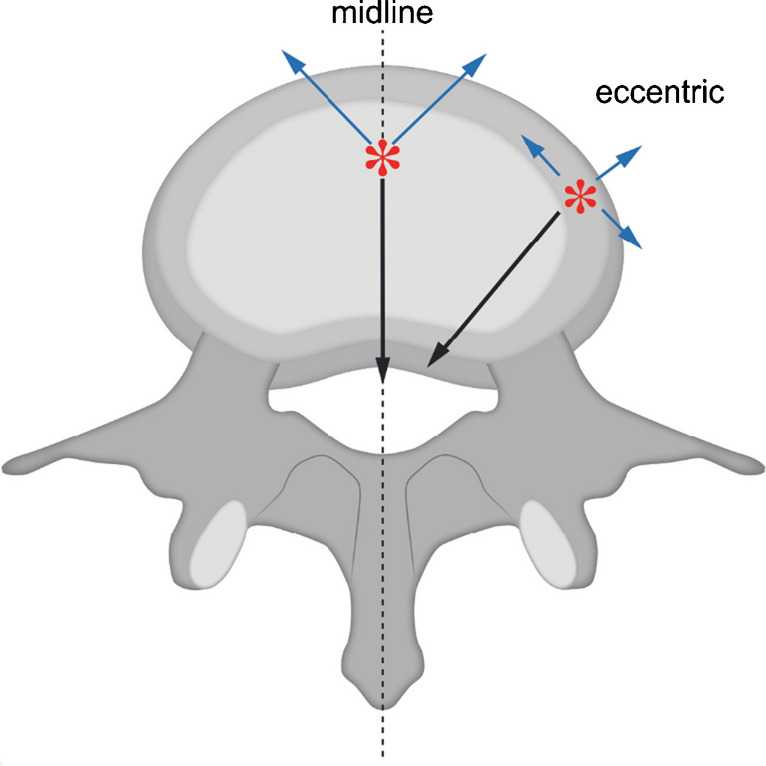
Graphic example of *midline* and *eccentric* location of the tumor epicenter (red asterisk), and tumor extension into the spinal canal—*intraspinal* (black arrows) and into the adjacent soft tissue—*extraspinal* (blue arrows)

A musculoskeletal radiologist (AN-15 years of experience) and a head-neck and neuroradiologist (BV-22 years of experience), blinded to all patient data, evaluated together all the scans for following parameters:

- Tumor origin was assessed on T1 and T2 MRI sequences and described as primary *midline* or primary *eccentric* (“*off midline*”) related to the epicenter of the mass. (Fig. 2)
- For tumors located in spine and sacrum, extension into the spinal canal or into the adjacent soft tissue was described as *intraspinal* and/or *extraspinal*, respectively. (Fig. 2)
- Bone destruction was evaluated on CT and classified as *geographical* or *permeative***/***moth-eaten*.
- Cortex destruction was assessed as *absent* or *present* on CT.
- Calcification was scored based on CT scans as absent or present and characterized as *rings-and-arcs/popcorn* (cartilaginous), *dense sclerotic* (osteoid) or *amorphous* (referring to bone remnant).
- Tumor shape was assessed on T2 fat-saturated MRI sequence as *lobulated* or *smooth*.
- Presence of cystic components on T1 and T2 MRI sequences, defined as fluid-like signal intensity components, was assessed as *single cyst* or *multicystic*. Presence of fluid–fluid levels in the cystic components was also recorded (*ABC*-like changes).
- Tumor signal intensity (SI) on water reconstruction of mDixon T2 and T1-TSE sequences was classified in three groups as: predominantly *low* (hypointense), *equal* (isointense) or *high* (hyperintense), relative to that of muscle.
- Amount of contrast enhancement (CE) on T1 fat-saturated post-contrast MRI was classified in two groups: *low-intermediate* (1–60%) and *high* (61–100%) depending on the amplitude of the predominant contrast enhancement within the lesion. Pattern of CE was defined as: *reticular* (marked enhancement in which several small linear structures are seen resembling a net-like aspect), *septo-nodular* (enhancement of small nodules and curved lines), *homogenous* (marked enhancement of homogeneous type without defining structures) or *inhomogeneous* (a mixture of enhancing and non- or less enhancing areas without defined pattern). Only the most dominant pattern of CE for each type of tumor was recorded.
- Functional MRI parameters of perfusion, permeability and diffusion were analyzed using *IntelliSpace Portal* (ISP) software (version 10.1, Philips, Best, The Netherlands). Total of 4 circular regions of interest (ROI) were drawn per tumor; each ROI was drawn on a different non-adjacent scan slice. Mean value of each of the following functional parameters measured within the ROI was calculated per histopathological group: *relative enhancement* – signal enhancement of a pixel of a certain dynamic relative to its pre-contrast (reference) dynamic, *maximal enhancement* – difference between average baseline SI and peak SI, *maximal relative enhancement* – maximum of all relative enhancements over all dynamics, *T0*—time at which SI increases at least 20% compared to the baseline SI, *time to peak*—time till contrast agent bolus reaches peak intensity, *wash in rate (WIR)* – maximum curve slope between T0 and time of SI peak (T1), *wash out rate (WOR)* – maximum curve slope between T1 and end of the measurement, *brevity of enhancement* – time between maximum WIR and maximum WOR; *Ktrans*—transfer constant between blood plasma and extravascular extracellular space (EES), *Kep* – efflux rate constant between EES and blood plasma, *Ve* – EES volume fraction, *Vp* – plasma volume fraction and *ADC* – apparent diffusion coefficient portraying the diffusion of water molecules within a tissue [18].

Perfusion time intensity curves (TIC) were analyzed per histopathological group and defined as *Type I* (no enhancement), *Type II* (persistent gradual increase of enhancement), *Type III* (rapid initial enhancement followed by a plateau phase), *Type IV* (rapid initial enhancement followed by a wash out phase) and *Type V* (rapid initial enhancement followed by sustained slower enhancement) [19].

Ultimately, after consensus of both radiologists (AN and BV), a radiological differential diagnosis was made based on the observed morphological parameters and compared to the histopathological diagnosis. When the radiological diagnosis was incorrect, we analyzed if functional parameters could have added value.

### Statistical analysis

Statistical analysis was done in RStudio (RStudio PBC, MA, USA, v1.4.1717). Differences between median ranks of functional parameters of different histopathological groups were analyzed using Dunn’s statistical test (α = 0.05) with Benjamini–Hochberg post hoc verification method.

## Results

### Epidemiology

Six different histopathological diagnoses were identified in this cohort, with chordoma being the most common (48/80 patients, 60.0%), followed by chondrosarcoma (13/80 patients, 16.3%), giant cell tumor of the bone (GCTB) (8/80 patients, 10.0%), plasmacytoma (7/80 patients, 8.7%), osteosarcoma (3/80 patients, 3.7%) and Ewing sarcoma (1/80 patients, 1.3%). We included one dedifferentiated chordoma of the skull base in the chordoma group. One GCTB located in the spine was malignant. One osteosarcoma of the mobile spine was periosteal, and two osteosarcomas located in the mobile spine and sacrum, respectively, were high grade osteosarcomas. Fifty out of 80 patients (62.5%) were male. Median age at diagnosis was 53.5 (range 18–85) with highest median age noted for chordoma (56.5) and lowest for Ewing sarcoma (18). Patient demographics is given in Table 1.

**Table 1:** Patient demographics

Diagnosis	Number of patients	Age (range)	Sex (M – male; F – female)
Chordoma	48	56.5 (22—85)	33 M; 15 F
Chondrosarcoma	13	42 (25–66)	4 M; 9 F
Giant cell tumor of the bone	8	42.5 (19—67)	3 M; 5 F
Plasmacytoma	7	53 (29—64)	6 M; 1 F
Osteosarcoma	3	45 (18 -56)	3 M
Ewing sarcoma	1	18	1 M

### Location and extension

Frequencies of different diagnoses per location are presented in Table 2. The only two tumor types located in the skull base in our series were chordoma (15/23 patients, 65.2%) and chondrosarcoma (8/23 patients, 34.8%). By far the most common sacral tumor was chordoma (25/36 patients, 69.4%). Other tumors were encountered in the mobile spine and sacrum with rather low frequency.

**Table 2:** Morphological imaging parameters of 80 patients in six different histopathological groups: chordoma, chondrosarcoma, plasmacytoma, giant cell tumor of the bone (GCTB), osteosarcoma and Ewing sarcoma

Location (number of patients)		Skull base (n = 23)		Mobile spine (n = 21)					Sacrum (n = 36)					
**Diagnosis**		Chordoma	Chondrosarcoma	Chordoma	Plasmacytoma	Giant cell tumor of the bone	Chondrosarcoma	Osteosarcoma	Chordoma	Giant cell tumor of the bone	Chondrosarcoma	Plasmacytoma	Ewing sarcoma	Osteosarcoma
**Number and (%) of patients per location**		15 (65.2%)	8 (34.8%)	8 (38.1%)	5 (23.8%)	4 (19.0%)	2 (9.5%)	2 (9.2%)	25 (69.4%)	4 (11.1%)	3 (8.3%)	2 (5.6%)	1 (2.8%)	1 (2.8%)
**Origin (*T1 and T2 MRI sequences*)**	Midline	13	1	8	5	3	1	2	25	3	1	1	1	0
	Eccentric	2	7	0	0	1	1	0	0	1	2	1	0	1
**Tumor extension (*T1 and T2 MRI sequences*)**	Intraspinal-extraspinal	NA	NA	4	2	1	1	0	18	1	0	2	1	1
	Intraspinal	NA	NA	1	2	1	1	1	3	2	0	0	0	0
	Extraspinal	NA	NA	3	0	1	0	1	4	1	2	0	0	0
**Bone destruction (*CT scan*)**	Geographical	14	8	2	5	3	1	1	19	4	1	2	0	1
	Permeated/moth-eaten	1	0	6	0	1*	1	1	5	0	2	0	1	0
**Cortex destruction (*CT scan*)**	absent	1	0	4	0	0	1	0	5	0	1	0	1	0
	present	14	8	4	5	4	1	2	18	4	2	2	0	1
**Calcification (*CT scan*)**	no calcification	3	1	6	5	4	0	0	9	4	0	2	1	0
	Rings-and-arcs/popcorn	0	7	0	0	0	2	0	0	0	3	0	0	0
	Dense sclerotic	0	0	0	0	0	0	2	0	0	0	0	0	1
	Amorphous	12	0	2	0	0	0	0	14	0	0	0	0	0
**Shape (*T2-fat saturated MRI sequence*)**	Lobulated	4	4	4	2	0	2	0	13	0	1	0	0	1
	Smooth	11	4	4	3	4	0	2	12	4	2	2	1	0
**Cystic components (*T1 and T2 MRI sequences*)**	no cystic components	15	8	7	5	4	2	1	25	2	3	2	1	0
	Single cyst	0	0	1	0	0	0	0	0	0	0	0	0	0
	Multicystic	0	0	0	0	0	0	1	0	2	0	0	0	1
**Signal intensity on T2-weighted MRI sequence**	Hypointense	0	1	0	1	0	0	1	0	2	0	0	0	0
	Isointense	0	0	0	0	0	0	1	0	0	0	0	0	0
	Hyperintense	15	7	8	4	4	2	0	25	2	3	2	1	1
**Signal intensity on T1-weighted MRI sequence**	Hypointense	2	2	3	1	4	0	2	19	4	3	0	1	0
	Isointense	1	1	1	0	0	0	0	3	0	0	0	0	1
	Hyperintense	12	5	4	4	0	2	0	3	0	0	2	0	0
**Contrast enhancement (*T1 fat-saturated post-contrast MRI*)**	1–60%	3	1	1	0	1	2	1	15	0	2	0	0	1
	61–100%	12	7	7	3	3	0	1	10	4	1	2	1	0
	Most common pattern	reticular	homogeneous	reticular	homogeneous	inhomogeneous	septo-nodular	inhomogeneous	reticular	inhomogeneous	septo-nodular	homogeneous	inhomogeneous	inhomogeneous

^*^—malignant GCTB, *NA* not applicable

Most tumors (64/80 patients, 80.0%) originated in the midline. (Table 2) These midline tumors were chordoma (46/64 patients 71.9%), plasmacytoma (6/64 patients 9.4%) and GCTB (6/64 patients 9.4%). At the level of mobile spine and sacrum, there were no major differences between midline and eccentric origin of various tumor types, with the exception of sacral chordoma. All 25 sacral chordomas originated in the midline. In the skull base only 9 tumors were eccentric (petroclival synchondrosis and/or petrous apex), 7 of these were chondrosarcomas (7/9 patients 77.8%). All but one of the midline skull base tumors were chordomas (13/14 patients, 93%).

Soft tissue extension of tumors located in spine or sacrum occurred in 54 patients. It was not seen in three small tumors (one spinal GCTB, one spinal plasmacytoma and one sacral chondrosarcoma). Combined *intra-* and *extraspinal* tumor extension was the most common (31/54 patients, 57.4%). (Table 2) *Intraspinal* or *extraspinal* extension only was seen in respectively 11 (20.4%) and 12 patients (22.2%). The contribution of chondrosarcomas to the combined *intra-* and *extraspinal* extension with only one patient (1/31, 3.2%) was small relative to the overall prevalence of chondrosarcomas. Of note, none of the six patients with plasmacytoma had *extraspinal* extension only.

### Bone destruction

**Fig. 3:**
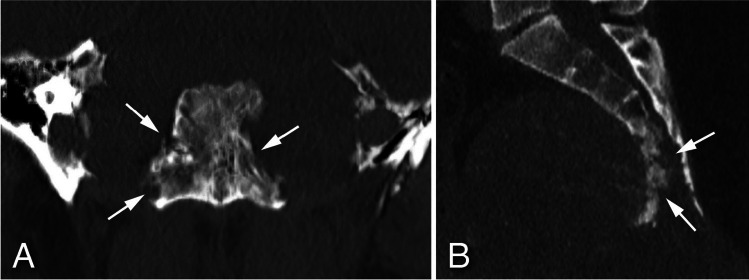
Examples of patterns of bone destruction: **A**- G*eographical* type of bone destruction pattern seen in a skull base chordoma centered dorsally in the clivus (arrows). extending into the apex os petrosum bilaterally. **B**- Example of *permeated/moth-eaten* type of bone destruction pattern seen in sacral chordoma (arrows)

*Geographical* bone destruction (Fig. 3A) was the most frequently seen pattern across all histopathological groups (61/78 patients, 78.2%), while *permeated/moth-eaten* type of bone destruction (Fig. 3B) occurred in 17 patients (21.8%), predominantly in the mobile spine and sacrum and only once in the skull base. (Table 2) *Permeated/moth-eaten* type of bone destruction was even the prevailing pattern in chordomas of the mobile spine (6/8 patients, 75.0%), and occurred in a few other tumor types including two of the three sacral chondrosarcomas and the malignant GCTB. This was also the only difference encountered between malignant and benign GCTB within this cohort. Destruction of cortex was common (65/78 patients, 83.3%) in all tumor types regardless of their location.

### Calcifications

**Fig. 4:**
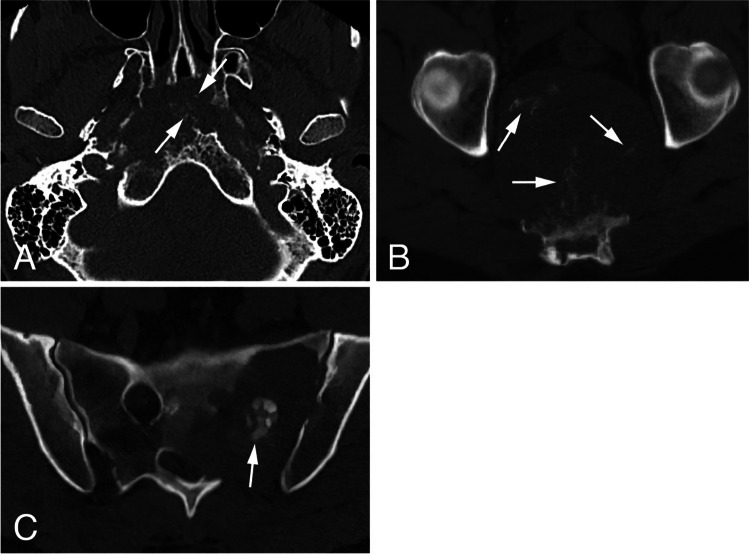
Examples of different calcification types: **A**- Axial view CT scan of a skull base chondrosarcoma showing *rings-and-arcs/popcorn*-like calcification (arrows). **B**- Axial view CT scan of sacral chordoma showing linear bone remnants (*amorphous*-like calcification) consistent with pre-existing bone (arrows). **C**- Axial view CT scan of sacral osteosarcoma presenting with *dense sclerotic*-like (osteoid) calcification (arrow)

*Rings-and-arcs/popcorn*-type calcification was seen on CT in 12 out of 78 patients (15.4%) all diagnosed with chondrosarcoma (Fig. 4A). *Amorphous-*type calcification (due to pre-existing bone) was seen in 28 patients out of 78 patients (35.9%), all diagnosed with chordoma—12 located in the skull base, 2 in the mobile spine and 14 in the sacrum (Fig. 4B). *Dense sclerotic*-type calcification was seen in only 3 patients (3.8%), all diagnosed with osteosarcoma (Fig. 4C). In 35 patients (44.9%) no calcification was present. (Table 2).

### Shape

Smooth margins occurred in the majority of skull base tumors (15/23 patients, 65.2%), especially among chordomas, in accordance with the prevalence of the histological diagnosis. (Table 2) As lobulated margins at the skull base were found in four patients with chordoma (4/8 patients, 50.0%) and in four chondrosarcoma patients, it was thus, relative to the histologic prevalence, more common in chondrosarcomas.

In the mobile spine, smooth margins were most common (13/21 patients, 61.9%), but were not present in the two chondrosarcomas.

**Fig. 5:**
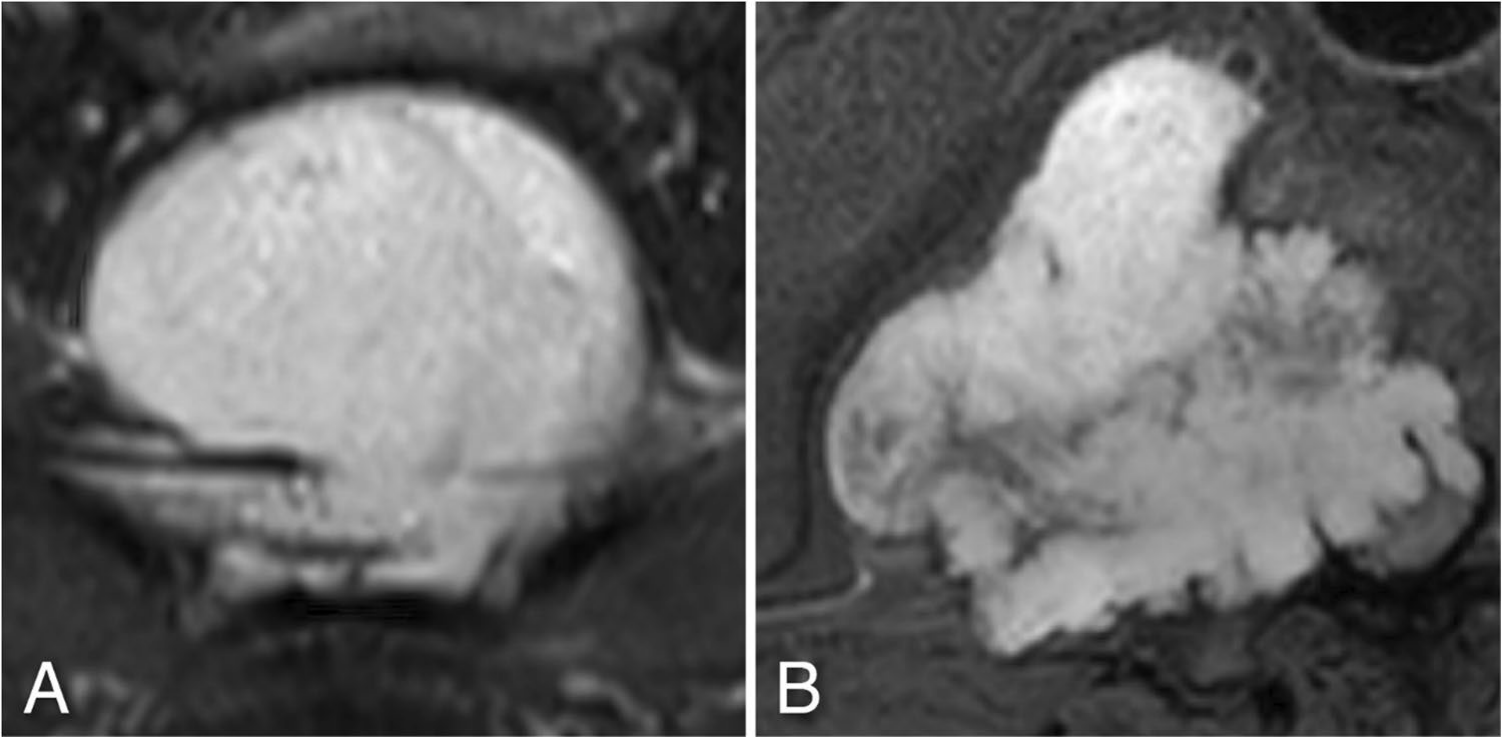
Examples of tumor shape. **A**- Smooth shape seen in a midline located chordoma of the sacrum with intraspinal-extraspinal extension on T2 fat-saturated MR image. **B**- Lobulated shape seen in an eccentrically located chondrosarcoma of the mobile spine with intraspinal-extraspinal extension on T2 fat-saturated MR image

Also in the sacrum, smooth margins were most common (21/36 patients, 58.3%), and seen in almost all histological diagnoses. Most patients had, in accordance with the histological prevalence, chordoma. (Fig. 5A) All four patients with GCTB had, as in the mobile spine, smooth margins. Lobulated margins were almost exclusively seen in chordomas (13/15 patients, 86.7%), only one chondrosarcoma and one osteosarcoma patient also had lobulated margins. (Fig. 5B).

### Cystic components

Cystic components were seen in only five out of 80 patients (6.3%). (Table 2) A *single cystic* component was seen in one patient with chordoma of the mobile spine. *Multicystic* changes were depicted in two patients with sacral GCTB and two with osteosarcoma. The cystic changes in the two sacral GCTB also had fluid–fluid levels consistent with *ABC*-like changes.

### Signal Intensity (SI)

**Fig. 6:**
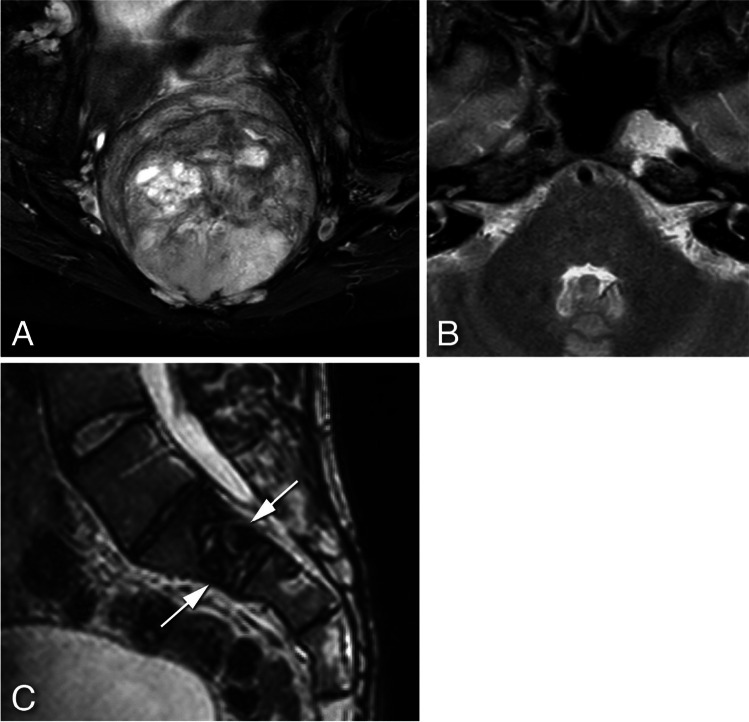
Examples of signal intensity. **A**- Heterogenous *hyperintensity* seen on T2-weighted MRI sequence in a midline located chordoma of the sacrum with extraspinal extension. **B**- Homogeneous *hyperintensity* seen on T2-weighted MRI sequence in chondrosarcoma of the skull base. **C**- *Hypointensity* seen on T2-weighted MRI sequence in a midline located sacral giant cell tumor of the bone with intraspinal extension

Predominantly *hyperintense* areas were visible on T2-weighted MR sequences in the majority of patients (74/80 patients, 92.5%), independent of histology and location. (Table 2) These areas appeared heterogeneous in 49 patients (Fig. 6A); distributed over chordomas (39/49 patients, 79.6%), GCTB (4/49 patients, 8.2%), chondrosarcoma (3/49 patients, 6.1%), and one each of osteosarcoma, plasmacytoma and Ewing sarcoma. These hyperintense areas appeared homogenous in the remaining 25 patients who had plasmacytoma (5/25 patients, 20.0%), chondrosarcoma (9/25 patients, 36.0%) (Fig. 6B), chordoma (9/25 patients, 36.0%) or GCTB (2/25 patients, 8.0%). *Hypointense* areas on T2-weighted sequences were seen in five tumors only; two of these were GCTB (Fig. 6C), and one each was chondrosarcoma, osteosarcoma and plasmacytoma. *Isointensity* was seen in only one patient with osteosarcoma.

On T1-weighted sequences *hypointense* areas were seen in 41 patients (51.3%) of which 24 had chordoma, 5 chondrosarcoma, 8 GCTB, 2 osteosarcoma, 1 plasmacytoma and 1 Ewing sarcoma. *Hyperintense* areas were seen in 32 patients (40.0%), out of which 19 had chordoma, 7 chondrosarcoma and 6 plasmacytoma. *Isointense* areas were seen in only 7 patients (8.7%) out of which 5 had chordoma, one chondrosarcoma and one osteosarcoma.

### Contrast Enhancement (CE)

Skull base tumors showed predominantly high CE (61–100%) independent of histology. In the mobile spine this was also the most common enhancement among all histologies, with the exception of the two chondrosarcomas who both displayed low-intermediate CE (1–61%). (Table 2) In the sacrum, low-intermediate CE was by far the most frequent in chordomas (15/18 patients, 83.3%). Most other tumors, with the exception of chondrosarcomas and osteosarcomas showed high CE (61–100%).

**Fig. 7:**
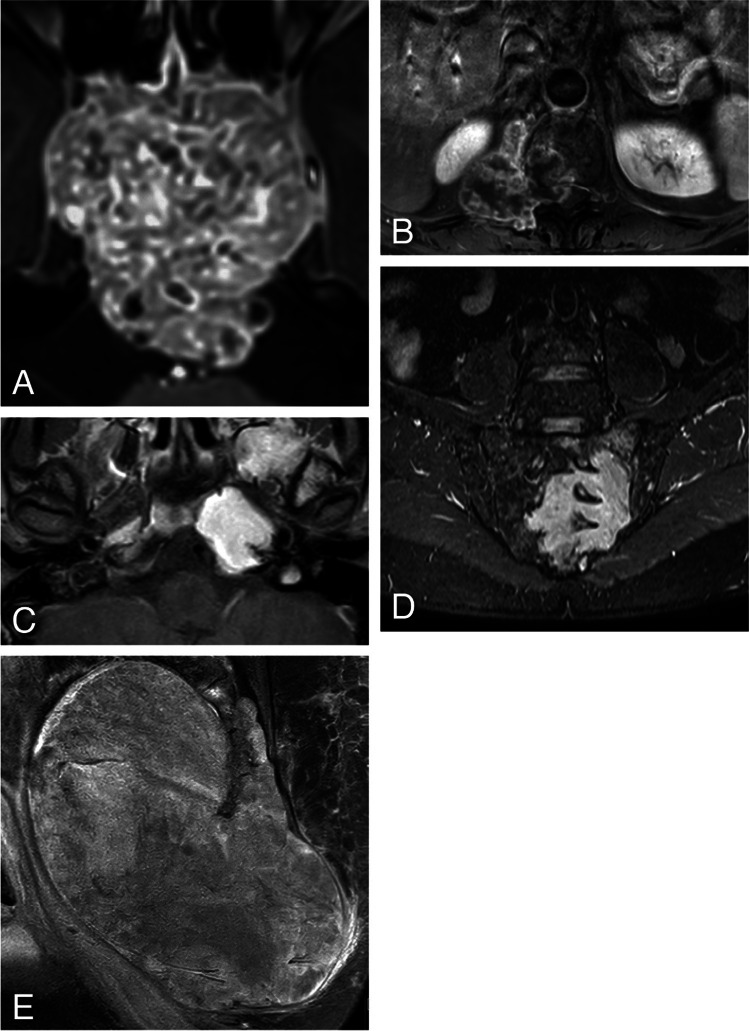
Examples of different types of contrast enhancement: **A** – *reticular* enhancement pattern in a midline located clival chordoma. **B** – *septo-nodular* enhancement pattern in an eccentrically located chondrosarcoma of the mobile spine with intraspinal-extraspinal extension. **C** – *homogeneous* enhancement type in chondrosarcoma located at the midline of the petroclival junction. **D** – *homogeneous* enhancement type in an eccentrically located sacral plasmacytoma with intraspinal-extraspinal extension. E – *inhomogeneous* enhancement type in a midline located sacral chordoma with intraspinal-extraspinal extension

*Reticular* CE pattern (Fig. 7A) was most commonly seen in chordoma patients, *septo-nodular* CE pattern (Fig. 7B) in patients with chondrosarcoma of the mobile spine and sacrum, and *homogeneous* CE pattern in patients with skull base chondrosarcoma (Fig. 7C) as well as in patients with plasmacytoma of the mobile spine and sacrum (Fig. 7D). *Inhomogeneous* CE (Fig. 7E) was seen in patients with chordoma, GCTB, osteosarcoma and Ewing sarcoma.

### MR perfusion

Perfusion analysis was performed in 73 patients. (Table 3) *Maximal enhancement* values of chordoma were significantly lower compared to those of chondrosarcoma (p = 0.046), GCTB (p = 0.044) and plasmacytoma (p = 0.005). *Maximal relative enhancement* (p = 0.009) and *wash in rate* values (p = 0.003) were significantly lower in chordoma compared to plasmacytoma. *Maximal relative enhancement* was also significantly lower (p = 0.013) in chordoma than GCTB. *Time to peak* values were significantly longer in both chordoma and chondrosarcoma compared to GCTB (p = 0.003; p = 0.004) and plasmacytoma (p = 0.004; p = 0.002). *Wash in rate* values of chondrosarcoma were significantly lower compared to plasmacytoma (p = 0.025).

**Table 3:** Mean values of perfusion parameters per histopathological diagnosis. Average area of the ROI – 93,50 mm^2^

Diagnosis	Number of analyzed patients	Relative enhancement [%]	Max enhancement	Max relative enhancement [%]	T0 [s]	Time to peak [s]	Wash in rate [s^−1^]	Wash out rate [s^−1^]	Brevity of enhancement [s]
Chordoma	45	81.17	380.65	76.54	56.66	225.15	38.87	24.65	92.40
Chondrosarcoma	12	96.64	987.71	157.52	46.23	223.64	109.77	39.74	61.66
Giant cell tumor of the bone	8	596.06	656.48	464.71	47.57	104.51	58.40	26.10	120.77
Plasmacytoma	5	99.61	1071.79	161.79	37.72	37.27	167.33	98.10	111.91
Osteosarcoma	2	72.81	370.75	88.27	25.40	168.79	42.87	21.56	190.28
Ewing sarcoma	1	72.62	283.11	81.55	20.29	282.62	29.42	15.50	190.06

**Fig. 8:**
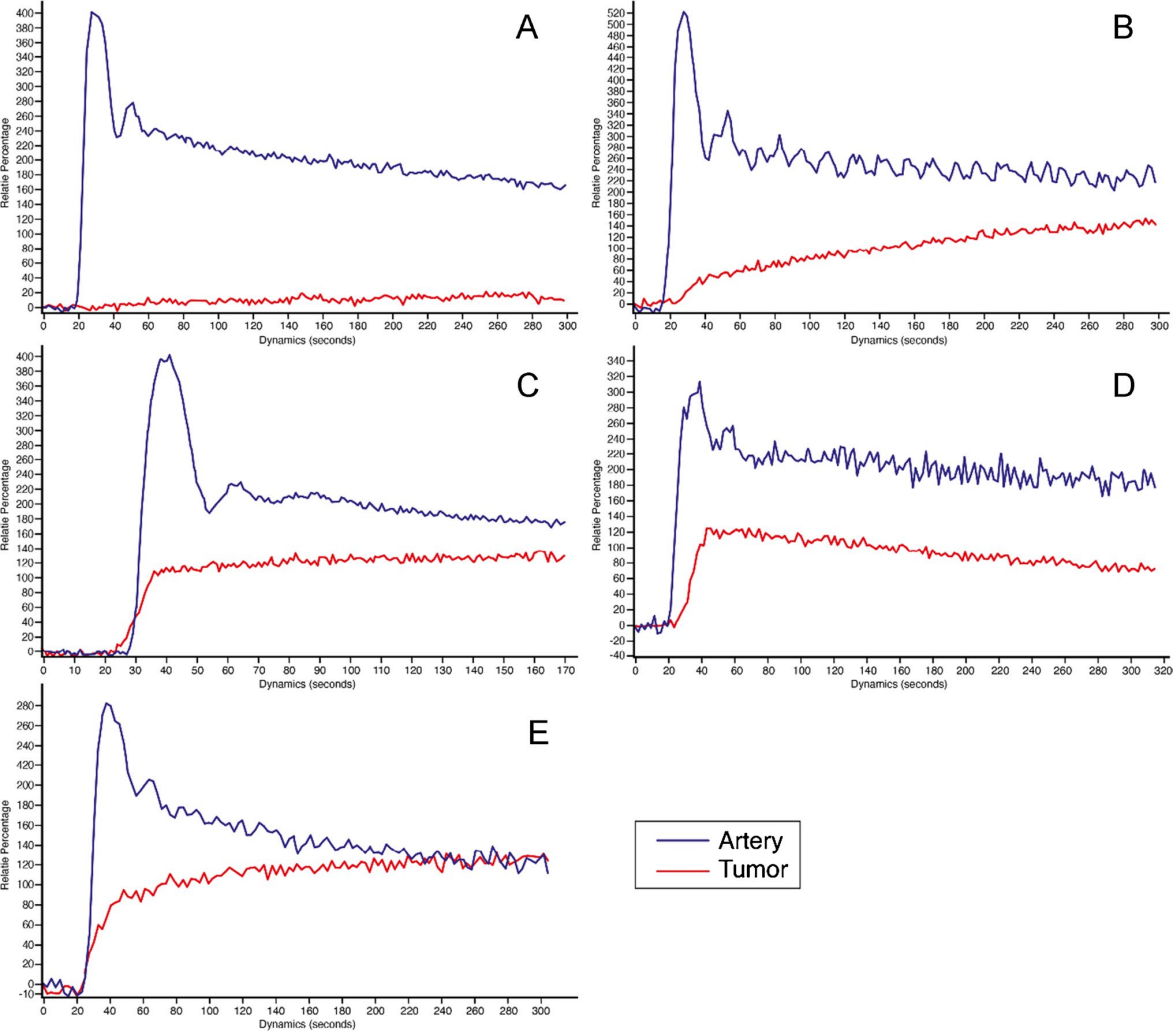
Time intensity curve (TIC) of the lesion and artery measured within the circular ROIs. **A**—Curve type I seen only in 8.2% of the patients, all diagnosed with chordoma – Example of a clival chordoma TIC. **B**—Curve type II seen in 19.2% of the patients, mostly chondrosarcoma and chordoma – Example of clival chondrosarcoma TIC. **C**—Curve type III seen in 35.6% of the patients, mostly chordoma, GCTB, osteosarcoma and chondrosarcoma—Example of clival chordoma TIC. **D**—Curve type IV seen in 13.7% of the patients, mostly plasmacytoma, including GCTB, chordoma and chondrosarcoma—Example of sacral plasmacytoma TIC. E—Curve type V seen in 23.3% of the patients, mostly chondrosarcoma, chordoma and Ewing sarcoma – Example of clival chondrosarcoma

Of the TIC’s, *Type I* was only seen in six patients with chordoma, and in no other tumor. (Fig. 8A) *Type II* curve was only seen in chordoma (9/14 patients, 64.3%), and chondrosarcoma (5/14 patients, 35.7%) (Fig. 8B). *Type III* curve was the most common one in chordoma (17/26 patients, 65.4%) (Fig. 8C), and GCTB (6/26 patients, 23.1%). It was also seen in two osteosarcomas, and one chondrosarcoma. *Type IV* curve was the only curve type seen in plasmacytoma (5/10 patients, 50.0%) (Fig. 8D); it was also encountered with lower frequencies in GCTB (2/10 patients, 20.0%), chordoma (2/10 patients, 20.0%), and chondrosarcoma (1/10 patients, 10.0%). *Type V* curve was seen in chondrosarcoma (5/17 patients, 29.4%) (Fig. 8E), chordoma (11/17 patients, 64.7%) and Ewing sarcoma (1/17 patients 5.9%).

### MR permeability

Permeability parameters were analyzed in 57 patients. (Table 4) Although V_p_ of chondrosarcoma was five times larger than that of chordoma, the parameters of chordoma and chondrosarcoma were not significantly different. However, K_trans_, K_ep_ and V_p_ were significantly lower in chordoma (p = 0.011; p = 0.004; p = 0.003) and chondrosarcoma (p = 0.014; p = 0.004; p = 0.007) compared to those of plasmacytoma. Values of K_trans_ and K_ep_ were significantly lower in chordoma ( p = 0.010; p = 0.011) and chondrosarcoma (p = 0.010; p = 0.006) compared to GCTB.

**Table 4:** Mean values of permeability parameters per histopathological diagnosis. Average area of the ROI—96,64 mm^2^

Diagnosis	Number of analyzed patients	K_trans_ [10^–3^/min]	K_ep_ [10^–3^/min]	V_e_ [10^–3^]	V_p_ [10^–3^]
Chordoma	36	43.17	219.23	282.71	4.10
Chondrosarcoma	8	34.20	109.01	247.57	21.99
Plasmacytoma	5	162.56	837.28	189.45	54.40
Giant cell tumor of the bone	5	178.79	682.88	380.00	30.98
Osteosarcoma	2	142.98	444.07	327.76	11.65
Ewing sarcoma	1	21.86	211.90	104.78	3.31

### Diffusion-weighted MRI

Overall chondrosarcoma had significantly higher ADC_min_ (p = 0.008), ADC_max_ (p = 0.017) and ADC_mean_ (p = 0.008) values compared to chordoma. (Table 5) Location wise this difference was most significant among the skull base tumors with p-values of < 0.001, 0.002and < 0.001 for ADC_min_, ADC_max_ and ADC_mean_, respectively. Single available cases of osteosarcoma, Ewing sarcoma, GCTB and plasmacytoma had lower ADC_mean_ values compared to chondrosarcoma—1.905·10^−3^mm^2^/s, 0.985·10^−3^mm^2^/s, 1.151·10^−3^mm^2^/s and 0.992·10^−3^mm^2^/s, respectively.

**Table 5:** Mean ADC values of CH and CS per location in 10^–3^ mm^2^/s. Average area of the ROI – 68.29 mm^2^

Diagnosis	Location	Number of analyzed patients	ADC_min_	ADC_max_	ADC_mean_
Chordoma	Clivus	9	1.287	1.882	1.573
	Mobile spine	1	1.085	1.680	1.360
	Sacrum	9	1.344	1.943	1.643
	Combined	19	1.303	1.900	1.595
Chondrosarcoma	Clivus	7	1.877	2.331	2.104
	Sacrum	1	1.915	2.595	2.347
	Combined	8	1.882	2.364	2.135

### Subjective final diagnosis

Overall, differential radiological diagnosis considered by the two reviewing radiologists, based on the morphological imaging parameters, coincided with the histopathological diagnosis in 72 patients (90%). In three patients (3.75%), two with chordoma and one with osteosarcoma, erroneous diagnoses of hemangioma, chondrosarcoma and GCTB were made. In 5 patients (6.25%) an unequivocal diagnosis could not be made. In two chordoma and one chondrosarcoma patients unequivocal diagnosis between chordoma and chondrosarcoma could not be made and, in two GCTB patients GCTB could not be differentiated from plasmacytoma.

Upon reviewing the functional imaging parameters of misdiagnosed patients, only 3 chordomas falsely diagnosed as chondrosarcoma could be considered as chordoma based on perfusion *wash in rate* parameter (21.87 s^−1^, 30.05 s^−1^, 50.84 s^−1^) and permeability V_p_ parameter (2.71·10^–3^, 7.09·10^–3^, 2.62·10^–3^) values which were approximate to the median values for chordoma presented in Tables 3 and 4.

## Discussion

The subjective diagnosis made by a musculoskeletal radiologist and a head-neck and neuroradiologist in consensus was accurate in 90% of the cases. Even this high accuracy can be improved when all parameters are combined, as is illustrated by the fact that in 3 out of the 8 misdiagnosed cases a correct diagnosis of chordoma instead of chondrosarcoma could have been made with the help of functional MRI parameters. The *wash in rate*s and V_p_ parameters were close to the mean values for chordoma and different from those of chondrosarcoma. As double reading of two highly specialized radiologists is not common practice, we tried to determine the relevance of morphological and functional imaging parameters, allowing a comprehensive diagnostic approach resulting in an objective, confident diagnosis with high accuracy in clinical practice.

In general, parameters in favor of chordoma are: midline location, *geographical* type of bone destruction in the skull base and sacrum and *permeated/moth-eaten* type of bone destruction in the spine, presence of *amorphous* calcifications, and *smooth* margins when located in the skull base; *hyperintense* heterogeneous SI on T2-weighted and *hypointense* SI on T1-weighted MRI sequences, *reticular* high CE pattern in the skull base and mobile spine and low-intermediate in the sacrum; low values of perfusion and permeability parameters such as *Type III* enhancement curve, low *max. enhancement*, low *wash in rate* (mean 38.87 s^−1^).

Parameters in favor of chondrosarcomas are: eccentric location, *geographical* type of bone destruction when located in the skull base, presence of *rings-and-arcs/popcorn*-type calcification, *lobulated* margin when located in the skull base and mobile spine, *hyperintense* homogeneous SI on T2-weighted and *hyperintense* SI on T1-weigted MRI sequences, *homogeneous* high CE pattern in the skull base and *septo-nodular* in the mobile spine and sacrum; high ADC values (mean 2.135·10^−3^mm^2^/s), higher values of perfusion and permeability parameters compared to those of chordoma, but lower than most other tumors, such as relatively high *max. enhancement*, and relatively high *wash in rate* (mean 109.77 s^−1^).

Parameters not in favor of chordoma or chondrosarcoma are: *smooth* margins at the sacral level (GCTB), presence of *multicystic* components or *ABC*-like changes (GCTB, osteosarcoma), *homogeneous* enhancement in the mobile spine and sacrum (plasmacytoma), high perfusion and permeability parameters (plasmacytoma, GCTB), such as high K_trans_, and K_ep_, short time to peak (GCTB), high wash in rate (plasmacytoma), high *max. enhancement* (GCTB, plasmacytoma), and high *max. relative enhancement* (GCTB).

Location is the first important parameter. When a primary bone tumor is suspected to be present in the axial spine, chordoma is a likely diagnosis. In our tertiary referral setting, 60% of the patients had chordoma. As this high prevalence obviously has an impact on frequency distribution of parameters, we compared these with the overall prevalence. When the tumor is located in the skull base, chordoma still has the highest prevalence, but chondrosarcoma is also frequent (34.8%) and is the most likely diagnosis when the tumor is located off-midline. Seven out of nine (77.8%) skull base tumors with such an eccentric location were chondrosarcoma. This can be explained by their mesenchymal cell/cartilaginous matrix root from the petroclival synchondrosis, as opposed to the predominantly midline chordoma emerging from the embryonic remnant of the primitive notochord (midline structure) [20]. Eccentric location of only two skull base chordoma is probably due to the presence of ectopic embryonic remnant notochordal tissue outside the notochordal path. All chordomas in the spine and sacrum were located in the midline, while chondrosarcoma in these locations occurred both in midline and eccentric locations.

In the spine and sacrum, the majority of tumors (57.4%) showed the combined *intra-* and *extraspinal* extension (Table 2). Because of the overall high prevalence of chordoma, and small numbers of other tumor types, chordoma was in almost each category of soft tissue extension the most prevalent. However, presence of *extraspinal* extension only, can be a useful parameter, since it was relatively rare (chordoma), or absent (plasmacytoma) in tumors originating in the midline. Conversely tumors with eccentric origin, such as chondrosarcoma, can show *extraspinal* extension only [3, 4, 7, 9, 11, 12].

Cortical destruction (83.3%), and *geographical* type (78.2%) of bone destruction (Fig. 3A) were common across all histopathological groups, and thus not highly discriminatory. Nevertheless, *permeated/moth-eaten* type bone destruction (Fig. 3B) was not uncommon in the mobile spine, including in chordoma (64.7%), which to our knowledge, hasn’t been reported before. Interestingly the only one of the three GCTB in the mobile spine with this *permeative*/*moth-eaten* pattern was the malignant one. This was at the same time the only difference between the benign and malignant GCTB. Obviously the *permeative*/*moth-eaten* pattern of bone destruction occurs in other malignancies, such as in non-sarcomatous tumors, and Ewing sarcoma and other small blue round cell sarcomas [21]. (Table 2).

Presence of *rings-and-arcs/popcorn*-like calcification, an indicator of a cartilaginous tumor, was seen on CT in all chondrosarcomas making this a beneficial feature in its diagnosis. The presence of *amorphous* calcification, representing bone remnant, was indicative of the chordoma diagnosis, as all patients with this type of calcification had a chordoma, located in decreasing order in the sacrum, skull base, and mobile spine. Unsurprisingly, *dense sclerotic*-type calcification was encountered in all three osteosarcoma patients [20].

So far, the relationship between tumor and margin type (*smooth* or *lobulated*) has not been reported in the literature. Even though the majority of tumors within this cohort showed *smooth* margins, a few deviations were noted. In the skull base half of the tumors with *lobulated* margins were chondrosarcomas, indicating that *lobulated* margins in this location are relatively more common in chondrosarcoma than in chordoma. At the level of mobile spine and sacrum chordoma was, in line with its high prevalence the dominant diagnosis when *lobulated* margins were present. In the category of *smooth* margins, the share of chordoma was less (12/34, patients 35.3%), mainly because of eight GCTB who all had *smooth* margins.

The presence of multicystic components normally indicates a diagnosis other than chordoma and chondrosarcoma. Not surprisingly we encountered multicystic changes in two patients with sacral GCTB (*ABC*-like changes) and in two out of three patients with osteosarcoma.

In the majority of patients (92.5%) tumors appeared *hyperintense* on T2-weighted sequences. Homogeneity was more frequent in plasmacytoma (20.0%) and chondrosarcoma (36.0%), relative to their general prevalence. The opposite was true in chordoma where homogeneity was lower (36.0%) than the general prevalence. On T1-weighted sequences chordoma tended to appear *hypointense* while chondrosarcoma tended to appear *hyperintense* regardless of their location, as reported elsewhere [3, 5–8, 10, 22]. This T1 hyperintensity in chondrosarcoma is likely to correlate with presence of mucoid/myxoid components. *Hyperintensity* seen among all the GCTB patients could correspond to bleeding as reported by *Si M. *et al. [4]

Most tumors (51/80 patients, 63.7%) showed high CE in the static phase, i.e., minutes after administration of the contrast agent. Only the majority of chondrosarcomas in the mobile spine, and chondrosarcomas and chordomas in the sacrum enhanced less (Table 2). Pattern and amount of tumor enhancement are directly related; the amount of tumor enhancement increases with tumor homogeneity. Plasmacytoma was the preferred diagnosis when the *homogeneous* pattern was seen in spine or sacrum (Fig. 7D) as all plasmacytomas presented with such enhancement. Chondrosarcoma located in the skull base was the only other tumor type that mainly had homogeneous enhancement. Interestingly the latter is different from what has been reported of chondrosarcoma in other parts of the skeleton. In our cohort, the chondrosarcoma located in spine and sacrum also displayed the well-known *septo-nodular* enhancement (Fig. 7B). The third type of enhancement that can be specific is the *reticular* type which is frequent in chordoma regardless of location (Fig. 7A). However, despite showing the same type of enhancement pattern, skull base chordomas showed higher CE compared to the sacral ones. High CE has been positively correlated with cellularity, which corresponds to the lower ADC values measured in the skull base chordomas of this cohort (Table 5) [25]. Furthermore, lower CE in sacral chordomas could be explained with presence of necrotic areas more commonly found in sacral than skull base chordomas [26].

The perfusion and permeability parameters of dynamic MRI characterize early enhancement prior to the static enhancement described in the previous paragraph. *Type III* and *IV* TICs, characterized by steep slopes followed by plateau or rapid wash-out phases are found in well perfused tumors that do not continue to capture contrast agent in a large interstitium [17]. Not surprisingly chordoma and plasmacytoma are main diagnoses with these two TIC types. Even though other tumors, such as GCTB, may also exhibit these types of TICs, chordoma should be considered once *Type III* TICs are encountered as shown in this cohort where the incidence of this TIC type in chordoma was greater than its histological prevalence [23]. The quantitative perfusion and permeability parameters may assist in further narrowing down of a differential diagnosis. In the well vascularized plasmacytoma and GCTB, K_trans_, and K_ep_ (for plasmacytoma also V_p_) are significantly higher, and time to peak shorter than in chordoma and chondrosarcoma [13]. The higher wash in rate of plasmacytoma did not reach a significant level compared to chordoma but did so compared to chondrosarcoma. Low capillary permeability and vascularization of chondrosarcoma portrayed by low K_trans_ and K_ep_ values in this cohort could be explained by the presence of myxoid [1]. Also, *maximal enhancement* and *maximal relative enhancement* are higher in plasmacytoma and GCTB than in chondrosarcoma and especially chordoma. M*aximal enhancement* of chordoma was significantly lower (p = 0.0462) compared to chondrosarcoma.

*Type II* and *V* curves indicate persistent capture of contrast agent over time after initial slow (*Type II*) or rapid (*Type V*) enhancement. While enhancement is limited early on, in the static phase marked enhancement is normally seen. This *Type II* curve was only seen in chordoma and chondrosarcoma. As described above, these tumors are, not surprisingly, also characterized by inhomogeneous, strong enhancement in the static phase. *Type I* curve meaning no enhancement at all, is also an indicative of chordoma, as it was only seen in chordoma, illustrating the many faces of this tumor. Such low enhancement has been previously reported in skull base chordomas*.* [24].

Despite being highly cellular, chondrosarcoma tend to have significantly higher ADC values compared to chordoma (Table 5) making this parameter, in combination with perfusion and permeability, a useful tool in differentiation. High ADC values in chondrosarcoma are most likely the reflection of their increased free extracellular water [12].

Main limitation of this study is a rather small sample size per histopathological group, other than chordoma. Therefore, an extended statistical analysis of the differentiating imaging features was unfortunately not feasible. Another limitation is the absence of metastases, especially in the mobile spine. Although a single osseous metastasis with soft tissue extension in a patient without a known primary neoplasm might have been included in our cohort. This did not happen because of population bias and because metastases are often multiple, and do not have a bulky soft tissue extension, especially, as seen in primary bone tumors of the sacrum. A final limitation is the participation of two specialized radiologists who scored in concert. This does not reflect common clinical practice. In other scenarios results, especially the final subjective diagnosis, probably would have been different. This underscores the need for quantifiable functional parameters.

## Conclusion

Expert radiologists are able to make an accurate subjective diagnosis of the rare primary osseous tumors in the axial skeleton, mainly based on morphological parameters. When no clear diagnosis can be made, morphological imaging parameters should be observed together with functional MRI parameters.

Diffusion MRI ADC values can help differentiate chordomas and chondrosarcomas. When TIC type III is encountered diagnosis of chordoma should be considered. Plasmacytoma and GTCB show higher Ktrans and Vp permeability values compared to chordoma and chondrosarcoma. Chordomas located in the mobile spine can show permeated/moth-eaten type of bone destruction. The only encountered difference observed between benign and malignant GCTB was the permeated/moth-eaten type of bone destruction in the latter.

## Data Availability

Due to the nature of the research, and corresponding rules of the local medial ethical committee supporting data is not available.

## Abbreviations

MRI: Magnetic resonance imaging
DCE: Dynamic contrast enhanced
TIC: Time intensity curve
CT: Computed tomography
GCTB: Giant cell tumor of the bone
CE: Contrast enhancement
